# Determination of the extent of dissection in early gastric cancer based on lymph node station power index

**DOI:** 10.1093/bjsopen/zrac104

**Published:** 2022-09-08

**Authors:** Chul-Hyo Jeon, Ki Bum Park, Sojung Kim, Ho Seok Seo, In-Ho Kim, Kyo Young Song, Han Hong Lee

**Affiliations:** Division of Gastrointestinal Surgery, Department of Surgery, Seoul St. Mary’s Hospital, College of Medicine, The Catholic University of Korea, Seoul, Republic of Korea; Division of Gastrointestinal Surgery, Department of Surgery, Seoul St. Mary’s Hospital, College of Medicine, The Catholic University of Korea, Seoul, Republic of Korea; Division of Gastrointestinal Surgery, Department of Surgery, Seoul St. Mary’s Hospital, College of Medicine, The Catholic University of Korea, Seoul, Republic of Korea; Division of Gastrointestinal Surgery, Department of Surgery, Seoul St. Mary’s Hospital, College of Medicine, The Catholic University of Korea, Seoul, Republic of Korea; Division of Medical Oncology, Department of Internal Medicine, Seoul St. Mary's Hospital, College of Medicine, The Catholic University of Korea, Seoul, Republic of Korea; Division of Gastrointestinal Surgery, Department of Surgery, Seoul St. Mary’s Hospital, College of Medicine, The Catholic University of Korea, Seoul, Republic of Korea; Division of Gastrointestinal Surgery, Department of Surgery, Seoul St. Mary’s Hospital, College of Medicine, The Catholic University of Korea, Seoul, Republic of Korea

## Abstract

**Background:**

The relative prognostic value of each lymph node (LN) station remains undefined in the treatment of gastric cancer. This study aimed to develop a new method to evaluate LN station ranking and define the optimal extent of lymphadenectomy for early gastric cancer.

**Methods:**

Clinical and histopathological information from patients who underwent curative gastrectomy with lymphadenectomy between 1989 and 2018 was reviewed. The LN station power index (LNPI) of each station was estimated using a LN retrieval frequency and the 5-year overall survival of patients with absence of LN at each station. External validation was conducted to evaluate the relevance of the LNPI.

**Results:**

A training set was developed from examination of 7009 patient records. For most nodal stations, the absence of LN was significantly associated with a poor prognosis. For the perigastric stations, the prognostic value assessed using the LNPI was in the following order: LN 4 (LNPI = 19.68), LN 3 (LNPI = 17.58), LN 6 (LNPI = 15.16), LN 1 (LNPI = 6.71), LN 2 (LNPI = 4.64) and LN 5 (LNPI = 2.86). The value rank of the extra-gastric stations was in the following order: LN 8a (LNPI = 12.93), LN 7 (LNPI = 10.51) and LN 9 (LNPI = 9.70), but the index of LN 12a (LNPI = 4.79) was higher than that of LN 11 (LNPI = 4.78). These trends in the LNPI were similar in the validation patient cohort.

**Conclusions:**

The LNPI is a simple tool to rank the priority of each LN station dissection. The optimal extent of D1 + lymphadenectomy using LNPI was determined to be D1 with LNs 7, 8a and 9.

## Introduction

Complications, survival, and recurrence following radical gastrectomy vary according to the extent of disease and lymphadenectomy. The Dutch D1D2 trial established that D2 lymphadenectomy (that included perigastric and extra-gastric nodes along the left gastric, splenic and hepatic arteries) decreased locoregional recurrence and gastric cancer-related death rates at the expense of greater postoperative mortality and complications compared with D1 dissection that encompassed only perigastric nodes^1^. Another study demonstrated that D2 nodal dissection, compared with D1 dissection, improves the survival rate of patients with gastric cancer if performed by experienced surgeons^2^. These results prompted studies on the extent of D1 and D2 lymphadenectomy, but none has investigated the relative prognostic value of each lymph node (LN) station.

The concept of D1+, where a limited dissection of extra-gastric nodes is performed, has been widely advocated in Korea and Japan for those patients with early gastric cancer (EGC) where curative endoscopic resection is considered unlikely^3,4^. The extent of D1 + dissection, however, remains undefined, and the optimal extent of the extra-gastric LN stations that should be included in D1+ lymphadenectomy has not been established.

This study aimed to evaluate the relative importance of each LN station in gastric cancer based on the LN collection using a new method, the LN station power index (LNPI), to define the optimal extent of D1+ lymphadenectomy for EGC.

## Methods

### Patient population

This study was approved by the Institutional Review Board of the College of Medicine, Catholic University of Korea (KC20RASI0180) in accordance with the Declaration of Helsinki and Good Clinical Practice. This study follows the STROBE reporting guideline^5^. Patients who underwent curative gastrectomy for gastric cancer between 1989 and 2018 at Seoul St. Mary’s Hospital were identified. Those with neoplasms other than gastric adenocarcinoma, gastric adenocarcinoma occurring in the remnant stomach, patients treated with neoadjuvant therapy or patients with missing operative and/or follow-up data were excluded. Clinicopathological data, including patient demographics, operative data, morbidity, tumour stage, postoperative recovery, recurrence and survival were collected. Preoperative clinical characteristics and postoperative complications were categorized according to the Eastern Cooperative Oncology Group (ECOG)^6^ and the Clavien–Dindo classifications^7^. Histological type was categorized as differentiated or undifferentiated. Poorly differentiated tubular adenocarcinoma, signet ring cell adenocarcinoma and mucinous adenocarcinoma were considered to be undifferentiated.

### Surgical details and histopathological analysis

Surgeons specializing in gastric surgery performed all operations based on Korean and Japanese gastric cancer treatment guidelines^3,4^. Specimens were removed by *en bloc* dissection and each LN was accurately mapped and collected intraoperatively or immediately after surgery via back-table dissection. Histology was reported by a team specializing in gastrointestinal neoplasia. Pathological results were reported in detail according to LN stations. Histological stage was determined based on the Eighth American Joint Committee on Cancer TNM criteria^8^.

### Follow-up

Regular follow-up programmes (every 3 months for advanced gastric cancer and 6 months for EGC), were followed for the first 3 years and annually thereafter. Follow-up included the determination of tumour marker levels, abdominal CT and endoscopic examination. Overall survival (OS) was calculated from the date of primary gastrectomy to the date of death from any cause or the time of the last follow-up.

### Definitions of LNPI

To evaluate LNPI, the frequency of absence of LN at each station was determined. Absence of LN was defined as the presence of only lymphatics and adipose or connective tissues without any pathological LNs despite accurate surgical dissection. Next, the cumulative 5-year OS rates of patients with no LNs retrieved at that station, irrespective of the presence/absence of retrieval at other stations, was calculated. The frequency of LN absence was derived from the number of patients without nodal tissue collection over the total number of dissected specimens. The index acquired by dissection of each station was calculated by multiplying the reciprocal of the LN retrieval frequency and the reciprocal of the 5-year survival rate of patients with LN absence multiplied by 100. The formula of the LNPI is as follows:

LNPI=NumberoftotaldissectedpatientsNumberofpatientswithLNabsence×1005−yearoverallsurvivalrateofpatientswithLNabsence

### External validation

External validation was conducted using data from St. Vincent Hospital, another tertiary hospital of this medical centre. The validation cohort included patients with gastric cancer who underwent curative gastrectomy from 2006 to 2015. The LNPI values of 1320 patients who were included according to the same criteria applied for the initial cohort were analysed. There was no difference in the surgeons’ experience level, operating method or follow-up protocol between the two institutions.

### Statistical analysis

Categorical variables were analysed using the chi-square test or Fisher’s exact test, as appropriate. Survival curves were constructed using the Kaplan–Meier method and differences in survival were compared using the log rank test. Cox proportional hazards regression models were used to calculate hazard ratios and 95 per cent confidence intervals (c.i.). All statistical analyses were performed using SPSS^®^ version 24.0 for Windows (IBM, Armonk, New York, USA). *P* < 0.050 was considered statistically significant.

## Results

### Patient demographics and clinicopathological characteristics

The initial cohort included 7009 patients and the validation cohort 1320. The median follow-up interval was 86.95 (range, 1–362) months. *Table 1* presents the general clinicopathological characteristics of the training cohort, in which patients were younger, underwent more limited lymphadenectomy (less than D2) and where the proportion of patients with upper gastric cancers and undifferentiated tumours was higher than that of the validation cohort. The 5-year OS rates of the training and validation groups were 77.3 per cent and 79.5 respectively (*P* = 0.195) (*Fig. S1*).

**Table 1:** Demographic and clinical variables in the training and validation cohorts

Variables	Training cohort (*n* = 7009)	Validation cohort (*n* = 1320)	*P*
**Age (years)**			0.001
<60	3466 (49.5)	540 (40.9)	
≥60	3543 (50.5)	780 (59.1)	
**Sex ratio (M:F)**			0.173
Male	4661 (66.5)	852 (64.5)	
Female	2348 (33.5)	468 (35.5)	
**BMI (kg/m^2^)**			0.776
<25	2244 (69.0)	879 (68.6)	
≥25	1007 (31.0)	403 (31.4)	
**ECOG**			0.247
0	3398 (49.1)	360 (47.7)	
1	3297 (47.6)	648 (49.1)	
2	208 (3.0)	34 (2.6)	
3	22 (0.3)	8 (0.6)	
**Resection**			0.080
TG	1819 (26.0)	312 (23.6)	
STG	5190 (74.0)	1008 (76.4)	
**Operating method**			0.477
Open	4487 (64.0)	745 (56.4)	
MIS	2100 (30)	575 (43.6)	
Others	422 (6.0)		
**LN dissection**			0.001
<D2	2332 (33.3)	346 (26.2)	
≥D2	4677 (66.7)	974 (73.8)	
**Tumour location**			0.001
Upper	885 (12.6)	110 (8.5)	
Middle and lower	6032 (86.1)	1166 (90.6)	
Others	92 (1.3)	114 (0.9)	
**Differentiation**			0.001
Differentiated	3156 (45.0)	667 (50.5)	
Undifferentiated	3853 (55.0)	653 (49.5)	
**pT category**			0.531
T1	3802 (54.2)	713 (54.0)	
T2	783 (11.2)	152 (11.5)	
T3	1062 (15.2)	216 (16.4)	
T4	1362 (19.4)	239 (18.1)	
**pN category**			0.235
N0	4268 (60.9)	841 (63.7)	
N1	874 (12.5)	155 (11.7)	
N2	698 (10.0)	128 (9.7)	
N3	1169 (16.7)	196 (14.9)	
**pTNM stage**			0.001
I	4043 (57.7)	773 (58.6)	
II	1149 (16.4)	233 (17.7)	
III	1449 (20.7)	281 (21.3)	
IV	368 (5.3)	33 (2.5)	

Values are *n* (%). Chi-square test was used to evaluate between-group differences in categorical variables and *P* < 0.050 was statistically significant. BMI, body mass index; ECOG, Eastern Cooperative Oncology Group performance status; LN, lymph node; MIS, minimally invasive surgery; STG, subtotal gastrectomy; TG, total gastrectomy.

### Correlations of LN absence with survival

**Fig. 1:**
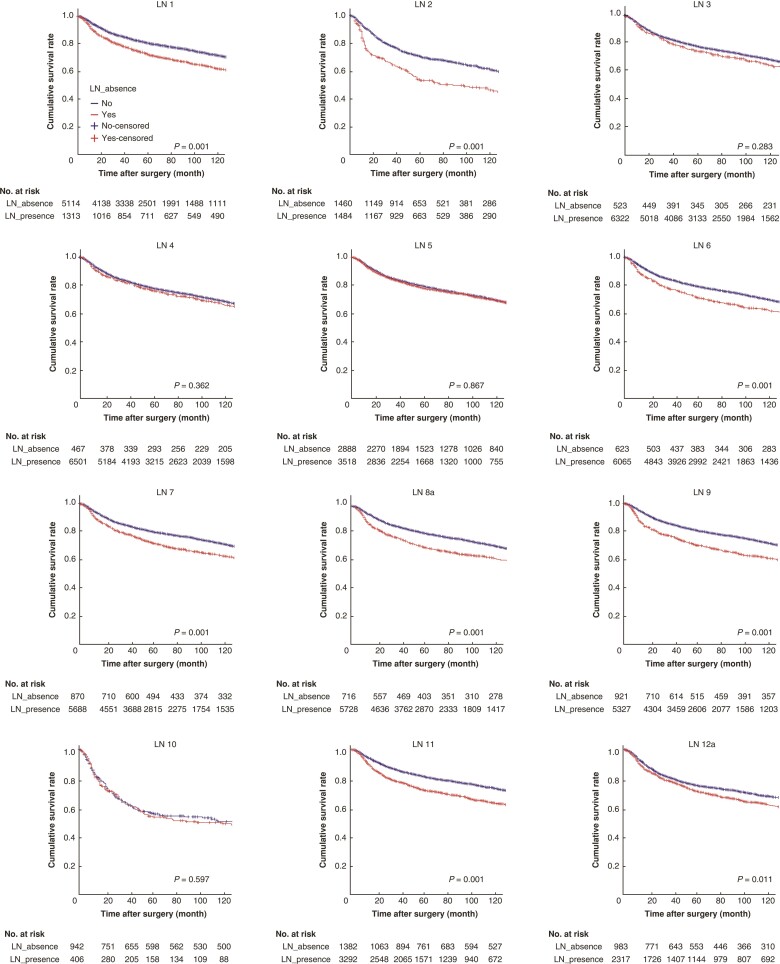
Kaplan–Meier survival curves of each nodal station of the training cohort

The status of LN absence and presence are summarized in *Table 2*. Total dissected refers to the number of patients where a LN was identified and dissected at that station. The highest was LN 4 with 7000 patients, while the lowest was LN 10 with 1356 patients. The survival rates of patients with LN presence ranged from 54.2 per cent to 80.7 per cent depending on station, and those of patients with LN absence ranged from 52.1 per cent to 77.6 per cent. The cumulative survival rate of all patients revealed that OS was significantly worse in patients with LN absence than patients with LN presence in most nodal stations, including LNs 1, 2, 6, 7, 8a, 9, 11 and 12a (*Fig. 1* and *Table 2*). At LN stations 3, 4, 5 and 10, there was no statistical difference between the presence of LN collection and OS (*Fig. 1* and *Table 2*).

The overall survival of all patients of the training cohort was significantly worse in patients with lymph node (LN) absence than patients with LN presence in most nodal stations, including LNs 1, 2, 6, 7, 8a, 9, 11 and 12a. In LNs 3, 4, 5 and 10, there was no statistical difference.

**Table 2:** The lymph node absence status in the training cohort

LN station	No. of total dissected cases	No. of patients with LN presence	5-year OS of patients with LN presence (%)	No. of patients with LN absence	5-year OS of patients with LN absence (%)	*P*
1	6454	5136	80.6	1318	72.5	0.001
2	2503	1492	70.6	1011	53.3	0.001
3	6875	6348	77.7	527	74.2	0.283
4	7000	6532	77.7	468	76.0	0.362
5	6433	3534	78.7	2899	77.6	0.867
6	6717	6091	78.9	626	70.8	0.001
7	6584	5708	79.9	876	71.5	0.001
8a	6470	5752	80.0	718	69.7	0.001
9	6274	5350	80.7	924	70.0	0.001
10	1356	409	54.2	947	52.1	0.597
11	4695	3305	80.5	1390	70.6	0.001
12a	3316	2328	74.1	988	70.0	0.011

LN, lymph node; No, number; OS, overall survival.

### Utilization of the LNPI according to LN station

**Fig. 2:**
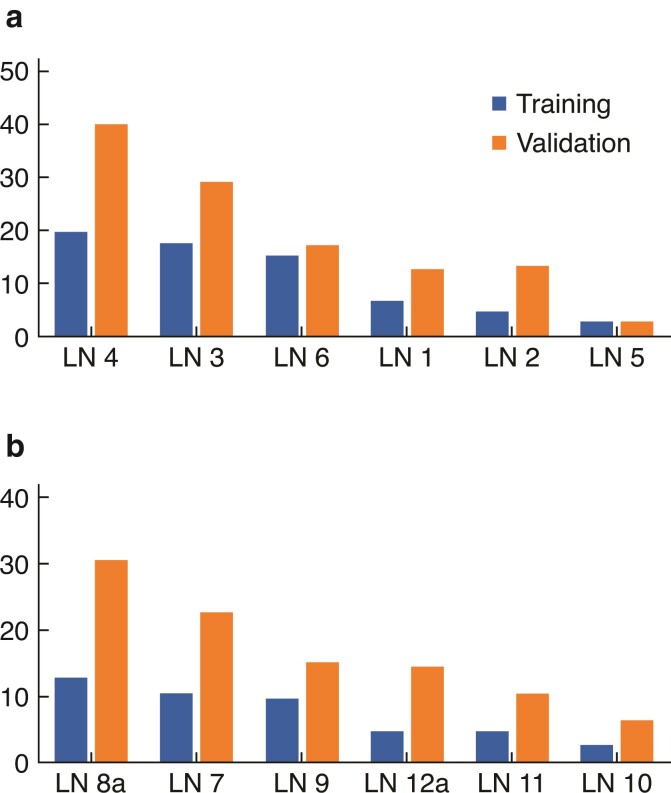
Lymph node station power index (LNPI) of each lymph node (LN) station

For the perigastric stations, the prognostic value assessed by the LNPI was in the following order: LN 4 (LNPI = 19.68), LN 3 (LNPI = 17.58), LN 6 (LNPI = 15.16), LN 1 (LNPI = 6.71), LN 2 (LNPI = 4.64) and LN 5 (LNPI = 2.86) (*Table 3* and *Fig. S2*). The results for the validation cohort were similar to the order of the training cohorts, except for a minimal difference between LNs 1 and 2 (*Fig. 2*). The index rank of the extra-gastric station was LN 8a (LNPI = 12.93), LN 7 (LNPI = 10.51), LN 9 (LNPI = 9.70), LN 12a (LNPI = 4.79), LN 11 (LNPI = 4.78) and LN 10 (2.75) (*Table 4* and *Fig. S2*). The order of the LNPI rank of the validation cohort for the extra-gastric stations was the same as with that of the training cohort (*Fig. 2*). Subgroup analysis was conducted according to the extent of resection (*Table S1*). The prognostic value of the extra-gastric station result was significantly higher in LNs 8a, 7 and 9 than in LN 11. Regardless of the extent of surgery and disease severity, the LNPI values of LNs 8a and 9 were significantly higher than those of LNs 11 and 12a (*Tables S1* and *S2*).

**a** LNPI of perigastric LN station. **b** LNPI of extra-gastric LN station. The LNPI of the validation cohort in the present study was also remarkably similar to the order of the training cohorts, except for a minimal difference between LNs 1 and 2. The order of the LNPI rank of the validation cohort for the extra-gastric station was the same as that of the training cohort.

**Table 3:** The lymph node station power index of the perigastric lymph node station in the training and validation cohorts

LN station	Training cohort			Validation cohort		
	Incidence of LN absence (%)	5-year OS (%)	LNPI	Incidence of LN absence (%)	5-year OS (%)	LNPI
1	20.42	72.5	6.71	11.76	66.8	12.73
2	40.39	53.3	4.64	12.32	60.6	13.40
3	7.67	74.2	17.58	4.64	74.0	29.14
4	6.69	76.0	19.68	3.82	65.6	39.88
5	45.07	77.6	2.86	45.91	81.3	2.68
6	9.32	70.8	15.16	6.56	89.2	17.10

LN, lymph node; LNPI, lymph node station power index; OS, overall survival.

**Table 4:** The lymph node station power index of the extra-gastric lymph node station in the training and validation cohorts

LN station	Training cohort			Validation cohort		
	Incidence of LN absence (%)	5-year OS (%)	LNPI	Incidence of LN absence (%)	5-year OS (%)	LNPI
7	13.31	71.5	10.51	5.28	83.2	22.75
8a	11.10	69.7	12.93	3.68	88.7	30.67
9	14.73	70.0	9.70	8.58	77.0	15.13
10	69.68	52.1	2.75	25.00	60.8	6.58
11	29.61	70.6	4.78	11.94	79.9	10.48
12a	29.80	70.0	4.79	7.98	86.1	14.55

LN, lymph node; LNPI, lymph node station power index; OS, overall survival.

## Discussion

The present study sought to understand the prognostic consequences of incomplete lymphadenectomy and develop a reliable index to help guide future lymphadenectomies during radical gastrectomy, The present study indicated that D1+ LN dissection would optimally include D1 nodes plus stations 7, 8a and 9.

LNPI offers an oncologic rank of each station, whether or not a node contains metastases. The results of the present study suggest priority of some LN stations. In this study, ‘LN absence’ was defined as having no retrieved pathological LN despite precise surgical dissection without violation of guidelines, irrespective of metastasis. This analysis hypothesized that OS was lower in the group with LN absence, regardless of tumour characteristics (location, size and depth of invasion), extent of surgery or disease stages. This relationship between LN absence and survival has been seen with in other malignancies. A study by Degiuli *et al.* confirmed that complete absence of nodes assessed was associated with a worse prognosis than node-negative and node-positive patients with rectal cancer who received neoadjuvant therapy^9^.

Regarding the concept of LN absence, numerous factors affect nodal retrieval including disease stage, the precise extent of dissection and the thoroughness of histopathological examination. At an accredited institution such as Seoul St. Mary’s Hospital, violation of operating principles is expected to be low^10^. The overall LN retrieval rate, including LN 7, was 58.5 per cent in previous studies evaluating lymphadenectomy in gastric cancer^11^ and others have reported numbers of retrieved LN as zero in many stations^12,13^. In a recent study about LN removal from individual stations, LN presence from station 7 was 77 per cent; compared with 87 per cent in the present study^14^. Similarly, the absence of LN in LN 5 was 61 per cent (1796 of 2932), compared with 45 per cent (2899 of 6433) in the present study^14^. Other studies have reported absence rates greater than 50 per cent for LN 5^11,13,15^.

There are some limitations to this study. Because of its single-centre retrospective nature, there are some unavoidable biases related to the long interval of observation (∼30 years) when therapeutic strategies may have changed. An optimal cut-off value for the LNPI remains unclear, although the use of external validation from another centre confirmed the significant results of the LNPI.

The LNPI is a useful method to measure and evaluate the relative prognostic value of LN stations related to radical gastrectomy regardless of cancer stage or extent of surgery. According to the results of this study, optimal D1+ is D1 lymphadenectomy with LNs 7, 8a and 9.

## Supplementary Material

zrac104_Supplementary_Data

## Data Availability

All data and materials are available upon request.
