# Supplementary material for: Determination of the extent of dissection in early gastric cancer based on lymph node station power index
Source: BJS Open. 2022 Sep 8;6(5):zrac104. doi: 10.1093/bjsopen/zrac104 (PMC9452541; doi:10.1093/bjsopen/zrac104)
Supplement: zrac104_Supplementary_Data [file zrac104_supplementary_data.docx]

Table S1 The LNPI of the LN station in the training cohorts according to the extent of resection

| LN station | Total gastrectomy | |  |  | LN station | Subtotal gastrectomy | | |
| --- | --- | --- | --- | --- | --- | --- | --- | --- |
|  | Incidence of  LN absence  (%) | 5-year OS  (%) | LNPI |  |  | Incidence of  LN absence  (%) | 5-year OS  (%) | LNPI |
| 3 | 5.65 | 63.3 | 27.97 |  | 4 | 6.85 | 78.30 | 18.65 |
| 6 | 7.15 | 55.5 | 25.21 |  | 3 | 8.35 | 76.60 | 15.64 |
|  |  |  |  |  |  |  |  |  |
| 4 | 6.22 | 67.4 | 23.86 |  | 6 | 10.04 | 74.30 | 13.41 |
| 8a | 9.22 | 48.4 | 22.42 |  | 8a | 11.71 | 75.20 | 11.35 |
| 9 | 11.71 | 53.0 | 16.11 |  | 7 | 13.72 | 75.30 | 9.68 |
| 7 | 12.08 | 58.50 | 14.16 |  | 9 | 15.73 | 74.20 | 8.57 |
| 2 | 15.48 | 53.30 | 12.12 |  | 1 | 20.58 | 76.40 | 6.36 |
| 1 | 19.96 | 60.50 | 8.28 |  | 12a | 30.29 | 73.10 | 4.52 |
| 11 | 25.61 | 58.30 | 6.70 |  | 11 | 31.16 | 74.60 | 4.30 |
| 12a | 28.30 | 30.30 | 5.86 |  | 5 | 44.03 | 81.40 | 2.79 |
| 10 | 38.72 | 52.10 | 4.96 |  |  |  |  |  |
| 5 | 48.19 | 67.30 | 3.08 |  |  |  |  |  |

LNPI indicates Lymph Node station Power Index; LN lymph node; OS overall survival.

Table S2 The LNPI of the LN station in the training cohorts in early and advanced gastric cancer

| Early gastric cancer | | | | | | | | Advanced gastric cancer | | | | | | | |
| --- | --- | --- | --- | --- | --- | --- | --- | --- | --- | --- | --- | --- | --- | --- | --- |
| LN  station | Total gastrectomy | | | LN  station | Subtotal gastrectomy | | | LN  station | Total gastrectomy | | | LN  station | Subtotal gastrectomy | | |
|  | Incidence of  LN absence  (%) | 5-year OS  (%) | LNPI |  | Incidence of  LN absence  (%) | 5-year OS  (%) | LNPI |  | Incidence of  LN absence  (%) | 5-year OS  (%) | LNPI |  | Incidence  of  LN absence  (%) | 5-year OS  (%) | LNPI |
| 6 | 4.76 | 86.7 | 24.22 | 4 | 6.10 | 94.0 | 17.44 | 3 | 6.01 | 47.0 | 35.39 | 4 | 8.16 | 58.1 | 21.10 |
| 8a | 5.12 | 85.8 | 22.78 | 3 | 7.53 | 90.2 | 14.73 | 4 | 5.74 | 55.8 | 31.23 | 3 | 9.76 | 58.8 | 17.42 |
| 3 | 5.09 | 93.8 | 20.93 | 6 | 8.68 | 90.7 | 12.70 | 6 | 8.76 | 43.4 | 26.30 | 6 | 12.42 | 54.3 | 14.82 |
| 4 | 6.91 | 83.1 | 17.41 | 8a | 10.10 | 94.9 | 10.43 | 8a | 12.00 | 37.7 | 22.10 | 8a | 14.54 | 52.0 | 13.22 |
| 9 | 7.95 | 88.2 | 14.26 | 7 | 12.15 | 93.3 | 8.82 | 7 | 13.58 | 41.4 | 17.79 | 7 | 16.55 | 52.1 | 11.60 |
| 7 | 9.91 | 93.1 | 10.84 | 9 | 12.94 | 93.7 | 8.25 | 9 | 14.39 | 39.7 | 17.50 | 9 | 20.71 | 53.0 | 9.11 |
| 2 | 12.10 | 86.1 | 9.60 | 1 | 17.17 | 93.5 | 6.23 | 2 | 17.92 | 38.5 | 14.49 | 1 | 26.88 | 57.2 | 6.50 |
| 1 | 17.05 | 92.7 | 6.33 | 11 | 27.38 | 92.7 | 3.94 | 1 | 22.06 | 43.5 | 10.42 | 12a | 30.02 | 55.3 | 6.02 |
| 11 | 19.96 | 91.8 | 5.46 | 12a | 30.56 | 91.3 | 3.58 | 12a | 26.83 | 47.3 | 7.88 | 11 | 36.80 | 55.5 | 4.90 |
| 12a | 31.93 | 87.0 | 3.60 | 5 | 44.44 | 94.8 | 2.37 | 11 | 29.16 | 44.1 | 7.78 | 5 | 43.29 | 57.7 | 4.00 |
| 10 | 40.77 | 91.0 | 2.70 |  |  |  |  | 10 | 38.06 | 38.6 | 6.81 |  |  |  |  |
| 5 | 48.77 | 93.7 | 2.19 |  |  |  |  | 5 | 47.79 | 49.4 | 4.24 |  |  |  |  |

LNPI indicates Lymph Node station Power Index; LN lymph node; OS overall survival.
